# *N*,*N*′-Bis(pyridin-4-ylmeth­yl)oxalamide benzene monosolvate: crystal structure, Hirshfeld surface analysis and computational study

**DOI:** 10.1107/S2056989019009551

**Published:** 2019-07-09

**Authors:** Sang Loon Tan, Nathan R. Halcovitch, Edward R. T. Tiekink

**Affiliations:** a Research Centre for Crystalline Materials, School of Science and Technology, Sunway University, 47500 Bandar Sunway, Selangor Darul Ehsan, Malaysia; b Department of Chemistry, Lancaster University, Lancaster LA1 4YB, United Kingdom

**Keywords:** crystal structure, bis­(4-pyridyl­meth­yl)oxalamide, benzene solvate, hydrogen bonding, Hirshfeld surface analysis, computational chemistry

## Abstract

The asymmetric unit of the title 1:1 solvate, C_14_H_14_N_4_O_2_·C_6_H_6_ [systematic name of the oxalamide mol­ecule: *N*,*N*′-bis­(pyridin-4-ylmeth­yl)ethanedi­amide], comprises a half mol­ecule of each constituent as each is disposed about a centre of inversion. In the oxalamide mol­ecule, the central C_2_N_2_O_2_ atoms are planar (r.m.s. deviation = 0.0006 Å). An intra­molecular amide-N—H⋯O(amide) hydrogen bond is evident, which gives rise to an *S*(5) loop. Overall, the mol­ecule adopts an anti­periplanar disposition of the pyridyl rings, and an orthogonal relationship is evident between the central plane and each terminal pyridyl ring [dihedral angle = 86.89 (3)°]. In the crystal, supra­molecular layers parallel to (10) are generated owing the formation of amide-N—H⋯N(pyrid­yl) hydrogen bonds. The layers stack encompassing benzene mol­ecules which provide the links between layers *via* methyl­ene-C—H⋯π(benzene) and benzene-C—H⋯π(pyrid­yl) inter­actions. The specified contacts are indicated in an analysis of the calculated Hirshfeld surfaces. The energy of stabilization provided by the conventional hydrogen bonding (approximately 40 kJ mol^−1^; electrostatic forces) is just over double that by the C—H⋯π contacts (dispersion forces).

## Abstract 2

The asymmetric unit of the title solvate comprises a half mol­ecule of each component as both species are disposed about a centre of inversion. In the crystal, two-dimensional arrays are formed by amide-N—H⋯N(pyrid­yl) hydrogen bonds, which are connected into a three-dimensional architecture by C—H⋯π(benzene and pyrid­yl) inter­actions with benzene acting as the acceptor and donor, respectively.

## Chemical context   

With a combination of centrally located amide and terminal pyridyl functional groups, the isomeric mol­ecules related to the title compound of the general formula (*n*-C_5_H_4_N)CH_2_N(H)C(=O)C(=O)N(H)CH_2_(C_5_H_4_N-*n*), for *n* = 2, 3 and 4, abbreviated as *^n^L*H_2_, have long attracted the attention of structural chemists and their structural chemistry has been reviewed very recently (Tiekink, 2017 ▸). Taking the ^3^ *L*H_2_ species as an exemplar, its 1:1 co-crystal with *N*,*N*′-di­carb­oxy­methyl­urea, HO_2_CCH_2_N(H)C(=O)N(H)CH_2_CO_2_H, features two distinct supra­molecular tapes sustained by N—H⋯O hydrogen bonding. The first of these arises from amide-N—H⋯O(amide) hydrogen bonding between the amide groups, on both sides of the ^3^ *L*H_2_ mol­ecule, through ten-membered amide synthons {⋯HNC_2_O}_2_ (Nguyen *et al.*, 2001 ▸). Parallel tapes comprising *N*,*N*′-di­carb­oxy­methyl­urea mol­ecules, sustained by six-membered {⋯O⋯HNCNH} synthons, are also formed. The links between the tapes leading to a two-dimensional array are of the type hy­droxy-O—H⋯N(pyrid­yl). Mol­ecules of *^n^L*H_2_ also featured prominently in early, systematic studies of halogen bonding. An illustrative example is found in the 1:1 co-crystal formed between ^3^ *L*H_2_ and 1,4-di-iodo­buta-1,3-diyne, I—C≡C—C≡C—C—I (Goroff *et al.*, 2005 ▸). A two-dimensional array is also found in this co-crystal whereby supra­molecular tapes between ^3^ *L*H_2_ mol­ecules are formed as for the previous example and these are connected by N⋯I halogen bonding. In the crystals of both polymorphs of pure ^3^ *L*H_2_ (Jotani *et al.*, 2016 ▸), similar supra­molecular tapes mediated by amide hydrogen bonding are formed. However, that this mode of supra­molecular association is not all pervasive in the *^n^L*H_2_ systems is seen the structures of the two polymorphs of pure ^4^ *L*H_2_ (Lee & Wang, 2007 ▸; Lee, 2010 ▸). In one of the polymorphs of this isomer, supra­molecular dimers are formed *via* amide-N—H⋯O(amide) hydrogen bonding and these are linked into a two-dimensional array *via* amide-N–H⋯N(pyrid­yl) hydrogen bonds (Lee & Wang, 2007 ▸). In the second polymorph, all potential amide-N—H and pyridyl-N donors and acceptors associate *via* amide-N–H⋯N(pyrid­yl) hydrogen bonds to generate a two-dimensional array. In this context, and in the context of recent work on ^4^ *L*H_2_ in co-crystals (Syed *et al.*, 2016 ▸) and adducts of zinc 1,1-di­thiol­ates (Arman *et al.*, 2018 ▸; Tan, Chun *et al.*, 2019 ▸), it was thought of inter­est to conduct a polymorph screen for ^4^ *L*H_2_. From a series of crystallizations of ^4^ *L*H_2_ taken in di­methyl­formamide and layered with benzene, *o*-xylene, *m*-xylene, *p*-xylene, toluene, pyridine and cyclo­hexane in separate experiments, only crystals of the title benzene solvate, (I), were isolated. Herein, the crystal and mol­ecular structures of (I) are described along with a further evaluation of the supra­molecular association *via* an analysis of the calculated Hirshfeld surfaces as well as a computational chemistry study.
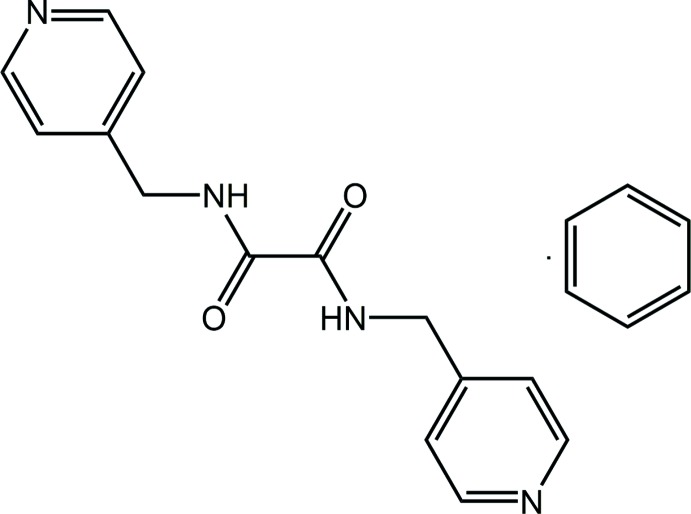


## Structural commentary   

**Figure 1:**
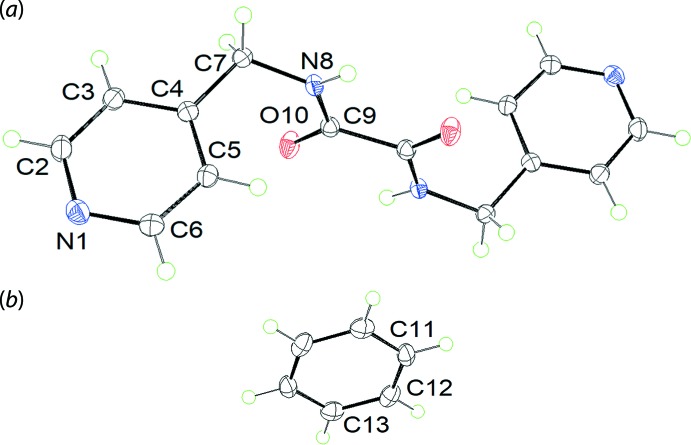
The mol­ecular structures of the constituents of the asymmetric unit of (I), showing the atom-labelling scheme and displacement ellipsoids at the 50% probability level. The mol­ecules are each disposed about a centre of inversion with the unlabelled atoms in (*a*) related by the symmetry operation: 1 − *x*, 1 − *y*, −*z* and those in (*b*) related by 1 − *x*, 1 − *y*, 1 − *z*.

The title co-crystal (I) is the result of crystallization of ^4^ *L*H_2_, taken in di­methyl­formaide, with benzene. The crystallographic asymmetric unit comprises half a mol­ecule each of ^4^ *L*H_2_ and benzene, Fig. 1 ▸, each being disposed about a crystallographic centre of inversion. The central C_2_N_2_O_2_ plane is strictly planar with the r.m.s. deviation of the fitted atoms being 0.0006 Å; the C7 atoms lie 0.0020 (16) Å to either side of the plane. An intra­molecular amide-N—H⋯O(amide)^i^ hydrogen bond, occurring between the symmetry related amide groups, gives rise to an *S*(5) loop, Table 1 ▸; symmetry operation (i) 1 − *x*, 1 − *y*, − *z*. The crystallographic symmetry also implies an anti­periplanar disposition of the pyridyl rings. The dihedral angle between the central plane and terminal pyridyl ring is 86.89 (3)°, indicating an orthogonal relationship.

## Supra­molecular features   

**Figure 2:**
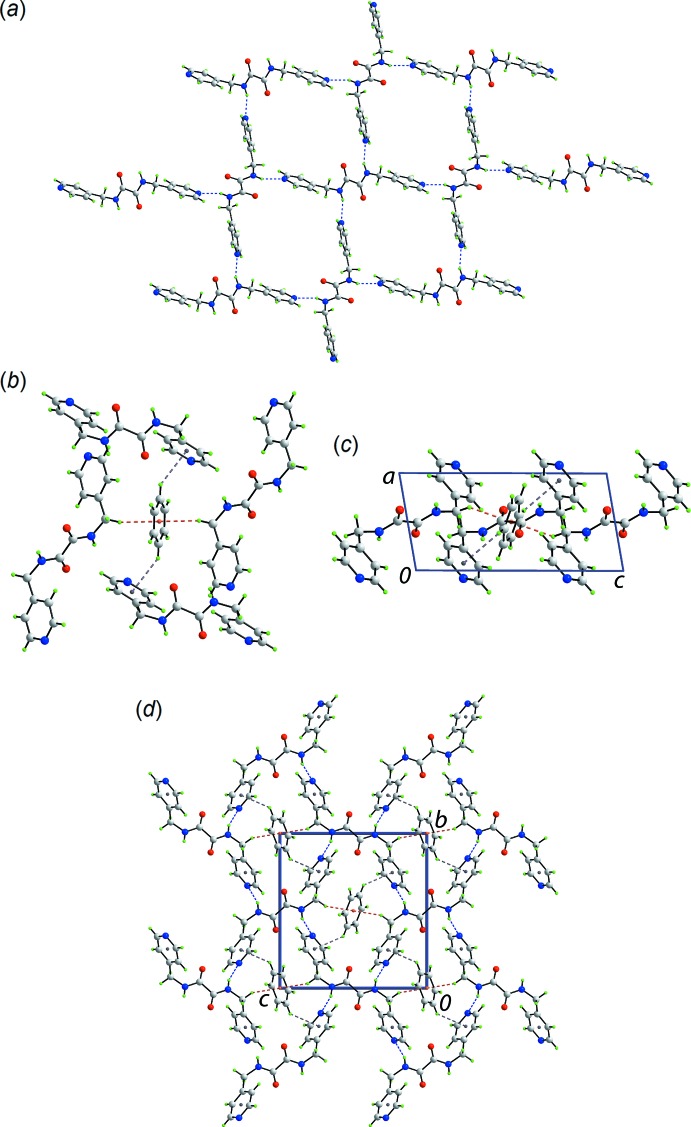
Mol­ecular packing in (I): (*a*) a view of the square grid sustained by amide-N—H⋯N(pyrid­yl) hydrogen bonding shown as blue dashed lines, (*b*) a view of the five-mol­ecule aggregate connected by methyl­ene-C—H⋯π(benzene) and benzene-C—H⋯π(pyrid­yl) inter­actions, shown as orange and purple dashed lines, respectively, (*c*) side-on view of the five-mol­ecule aggregate and (*d*) a view of the unit-cell contents shown in projection down the *a* axis.

The geometric parameters characterizing the inter­atomic contacts identified in the crystal of (I) are given in Table 1 ▸. The key feature of the mol­ecular packing is the formation of amide-N—H⋯N(pyrid­yl) hydrogen bonding. This generates a two-dimensional, rectangular grid lying parallel to (10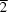), Fig. 2 ▸(*a*), with dimensions defined by O10⋯O10 and N8⋯N8 separations of 9.6770 (11) and 12.3255 (11) Å, respectively. The other notable contacts in the crystal are of the type C—H⋯π, Table 1 ▸. Thus, methyl­ene-C7—H⋯π(benzene) and benzene-C11—H⋯π(pyrid­yl) inter­actions are formed. From sym­metry, each benzene mol­ecule forms four, *i.e*. two (as acceptor) and two (as donor), such inter­actions, Fig. 2 ▸(*b*). The side-on view of Fig. 2 ▸(*b*) shown in Fig. 2 ▸(*c*) indicates the amide-N—H and pyridyl-N project in all directions around the five-mol­ecule aggregate. Indeed, it is the C—H⋯π inter­actions that connect the layers into a three-dimensional architecture, Fig. 2 ▸(*d*).

**Figure 3:**
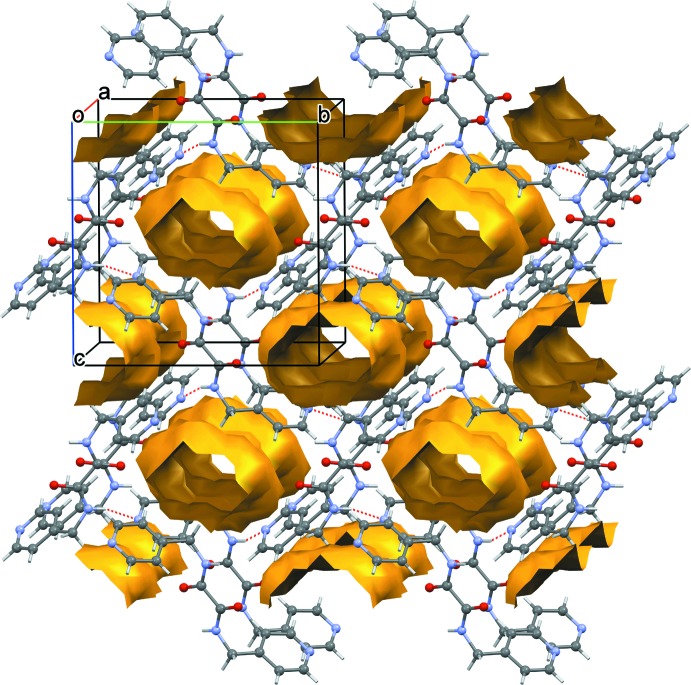
A plot of the solvent-accessible voids in the crystal of (I) upon removal of the solvent benzene mol­ecules within a 2 × 2 × 2 set of unit cells.

Upon removing the benzene mol­ecules within a 2 × 2 × 2 set of unit cells, the packing was subjected to a calculation of solvent-accessible void space in *Mercury* (Macrae *et al.*, 2006 ▸) with a probing radius of 1.2 Å. The results showed that the packing devoid of benzene comprises approximately 25.8% of the volume which is equivalent to 227.3 Å^3^ of void space, as illustrated in Fig. 3 ▸.

## Hirshfeld surface analysis and computational study   

To gain a better understanding of the nature of the inter­molecular inter­actions identified in (I), the overall structure of (I) as well as the individual ^4^ *L*H_2_ and benzene mol­ecules were subjected to a Hirshfeld surface analysis using *Crystal Explorer 17* (Turner *et al.*, 2017 ▸) based on the procedures as described in the literature (Tan, Jotani *et al.*, 2019 ▸).

**Figure 4:**
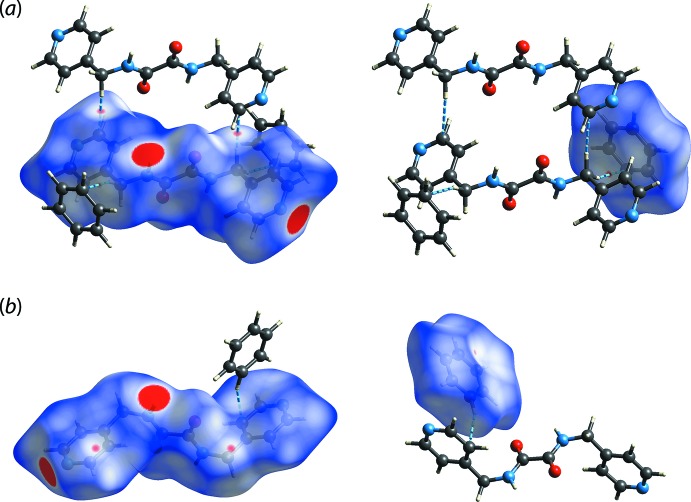
The *d*_norm_ maps within the range of −0.0567 to 0.9466 arbitrary units for the ^4^ *L*H_2_ (left) and benzene (right) mol­ecules: (*a*) highlighting the amide-N—H⋯N(pyrid­yl) (intense red) and methyl­ene-C7—H7*B*⋯π(benzene) (faint red) contacts with the intensity relative to the contact distance and (*b*) highlighting the connections between mol­ecules mediated by benzene-C11—H11⋯π(pyrid­yl) inter­actions.

The Hirshfeld surface mapped over *d*_norm_ map of ^4^ *L*H_2_ displays several red spots, that range from intense to weak, which reflect the inter­actions identified in the crystal (Spackman & Jayatilaka, 2009 ▸). The intense red spots arise from amide-N—H⋯N(pyrid­yl) hydrogen bonds while the diminutive spots originate from methyl­ene-C7—H7*B*⋯π(benzene) inter­actions, Fig. 4 ▸(*a*), with both indicative of contact distances shorter than the respective sum of the van der Waals radii. Reflecting the relatively long separation, the benzene-C11—H11⋯π(pyrid­yl) inter­action is reflected as only a white spot as the contact distance is only just within the sum of van der Waals radii, as shown in Fig. 4 ▸(*b*).

**Figure 5:**
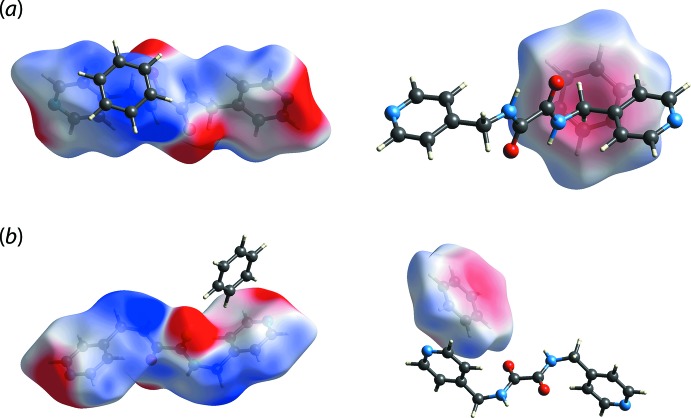
The calculated electrostatic potential mapped onto the Hirshfeld surfaces with the isosurface value range of −0.0257 to 0.0389 atomic unit for the ^4^LH_2_ (left) and benzene (right) mol­ecules showing the charge complementarity for the (*a*) methyl­ene-C7—H7*B*⋯π(benzene) and (*b*) benzene-C11—H11⋯π(pyrid­yl) inter­actions.

The C—H⋯π inter­actions were subjected to electrostatic potential mapping for verification purposes. The result shows that the methyl­ene-C7—H7*B*⋯π(benzene) contact is indeed electrostatic in nature as revealed by the distinct blue (*i.e*. electropositive) and red (*i.e*. electronegative) colour scheme on the surface of the contact points, Fig. 5 ▸(*a*). In contrast, the benzene-C11—H11⋯π(pyrid­yl) contact displays pale colouration around the contact zone suggesting that the inter­action could be attributed to weak dispersion forces, Fig. 5 ▸(*b*).

**Figure 6:**
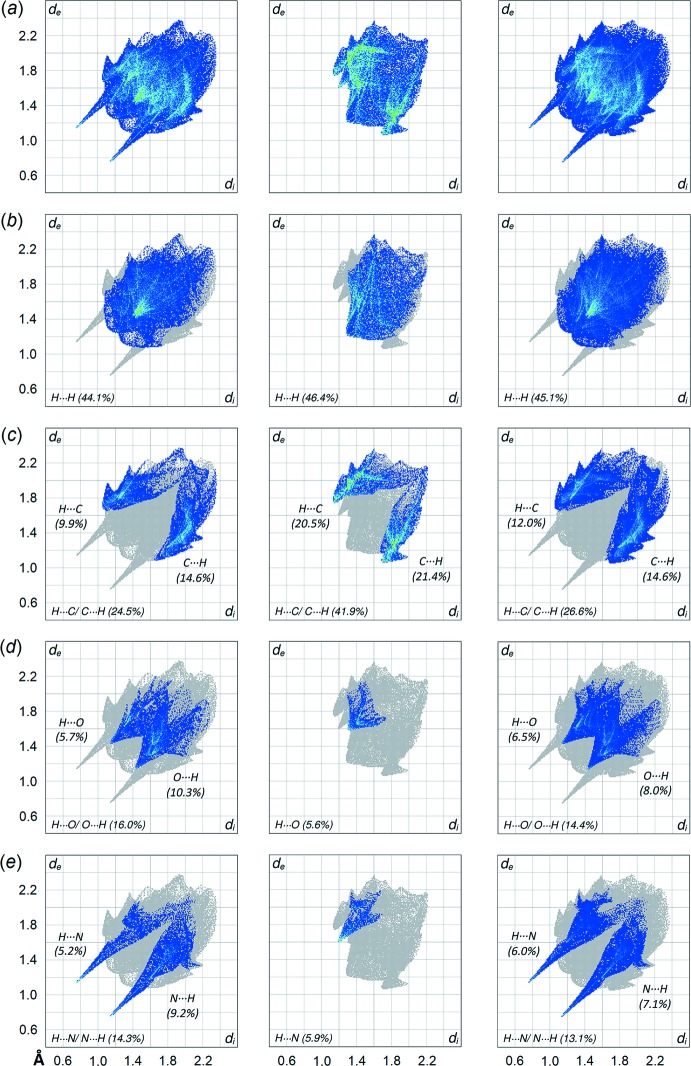
(*a*) The overall two-dimensional fingerprint plots for ^4^ *L*H_2_, benzene and overall (I), and those delineated into (*b*) H⋯H, (*c*) H⋯C/ C⋯H, (*d*) H⋯O/ O⋯H and (*e*) H⋯N/ N⋯H, with the percentage contribution being specified for each contact indicated therein.

The two-dimensional fingerprint plots were generated for overall (I) as well as its individual mol­ecules to qu­antify the close contacts identified through the Hirshfeld surface analysis, see Fig. 6 ▸(*a*)–(*e*). As shown in the overall fingerprint plot in Fig. 6 ▸(*a*), (I) exhibits a bug-like profile with distinctive symmetrical spikes which are similar to those exhibited by the individual ^4^ *L*H_2_ mol­ecule, therefore indicating that the inter­molecular inter­actions in (I) are mainly sustained by ^4^ *L*H_2_ mol­ecules. Decomposition of the overall fingerprint plots of (I) shows that the contacts are mainly dominated by H⋯H (45.1%; *d*_i_ + *d*_e_ ∼2.42 Å), H⋯C/C⋯H (26.6%; *d*_i_ + *d*_e_ ∼2.66 Å), H⋯O/O⋯H (14.4%; *d*_i_ + *d*_e_ ∼2.58 Å), H⋯N/N⋯H (13.1%; *d*_i_ + *d*_e_ ∼1.88 Å) and other contacts (0.8%). Except for the H⋯H contacts, to differing extents, the remaining major contacts are shorter than the corresponding sum of van der Waals radii for H⋯C (∼2.90 Å), H⋯O (∼2.72 Å) and H⋯N (∼2.75 Å).

The individual ^4^ *L*H_2_ mol­ecule exhibits at similar distribution of the major contacts compared to overall (I). However, some distinctions are observed on the external and inter­nal contacts upon further delineation of the corresponding decomposed fingerprint plots. While the distribution is rather symmetric in overall (I), for ^4^ *L*H_2_ these are either inclined towards the external or inter­nal contacts presumably due to inter­action with the solvent benzene mol­ecule. For instance, the H⋯C/C⋯H contact in the individual ^4^ *L*H_2_ mol­ecule comprises 9.9% (inter­nal)-H⋯C-(external) and 14.6% (inter­nal)-C⋯H-(external) contacts as compared to 12.0 and 14.6% for the equivalent contacts in overall (I), Fig. 6 ▸(*c*). Similar observations pertain for the H⋯O/ O⋯H and H⋯N/ N⋯H inter­actions, Fig. 6 ▸(*d*)–(*e*).

As for the benzene mol­ecule, an irregular fingerprint profile is noted with the distribution dominated by H⋯H (46.4%) and H⋯C/ C⋯H (41.9%) surface contacts. The latter are almost equally distributed between the inter­nal and external contacts, *i.e*. 20.5% for (inter­nal)-H⋯C-(external) and 21.4% for (inter­nal)-C⋯H-(external) contacts. In addition, the solvent mol­ecules are sustained in the mol­ecular architecture through minor contributions from H⋯O (5.6%) and H⋯N (5.9%) contacts, respectively. These inter­actions are at distances of ∼2.52 Å (H⋯H), ∼2.92 Å (H⋯C/C⋯H), ∼2.98 Å (H⋯O) and ∼2.79 Å (H⋯N), which are greater than the corresponding sum of van der Waals radii, indicating the identified C—H⋯π(benzene and pyrid­yl) inter­actions can largely be considered as localized inter­actions.

## Computational chemistry study   

**Figure 7:**
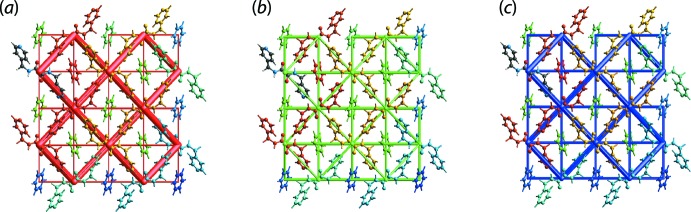
Energy framework of (I) as viewed down along the *a*-axis direction, showing the (*a*) electrostatic potential force, (*b*) dispersion force and (*c*) total energy diagrams. The cylindrical radii are proportional to the relative strength of the corresponding energies and they were adjusted to the same scale factor of 120 with a cut-off value of 5 kJ mol^−1^ within 2 × 2 × 2 unit cells.

The calculation of inter­action energy was performed using *Crystal Explorer 17* based on the procedures as described previously (Tan, Jotani *et al.*, 2019 ▸). As expected, the greatest inter­action energy in the crystal of (I) is found for the amide-N—H⋯N(pyrid­yl) contact having a total energy (*E*_int_) of −38.1 kJ mol^−1^, Table 2 ▸. This is followed by methyl­ene-C7—H7*B*⋯π(benzene) and benzene-C11—H11⋯π(pyrid­yl) contacts with a very similar *E*_int_ values of −18.9 and −16.9 kJ mol^−1^, respectively, despite the *d*_norm_ contact distance being significantly greater for the latter. The calculation results reveal that the repulsion energy is greater in methyl­ene-C7—H7*B*⋯π(benzene) compared with the benzene-C11—H11⋯π(pyrid­yl) contact, which contributes to the slight variation in their *E*_int_ values. In short, the N—H⋯N inter­action is stabilized largely by electrostatic forces while the C—H⋯π inter­actions are stabilized largely by dispersion forces. Overall, the crystal of (I) is dominated by electrostatic forces that form a cross-shaped energy framework that encompasses the void space in the unit cell. This framework is further stabilized by dispersion forces that co-exist within the void owing to the weaker inter­actions between the solvent mol­ecules with the host, Fig. 7 ▸(*a*)–(*c*).

**Figure 8:**
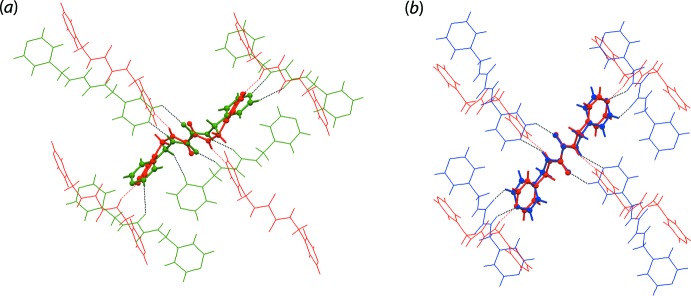
A comparison of mol­ecular packing of ^4^ *L*H_2_: (*a*) (I) (red image) and Form I (green) and (*b*) (I) (red) and Form II (blue), showing the differences between five pairs of ^4^ *L*H_2_ mol­ecules with an overall r.m.s. deviation of 0.587 and 0.403 Å, respectively.

Calculations were also performed to compare the mol­ecular packing similarity of (I) with the two polymorphic forms of ^4^ *L*H_2_ available in the literature (Lee & Wang, 2007 ▸; Lee, 2010 ▸). Mol­ecular clusters of (I), Form I and Form II containing 20 ^4^ *L*H_2_ mol­ecules each were subjected to mol­ecular packing analysis using *Mercury* (Macrae *et al.*, 2006 ▸), with the geometric tolerances being set to 20% (*i.e.* only molecules within the 20% tolerance for both distances and angles were included in the calculation and molecules with a variation >20% were discarded); molecular inversions were enabled during calculation. The result shows that out of the 20 mol­ecules in the cluster, only one ^4^ *L*H_2_ mol­ecule in each polymorph resembled the reference packing in (I) with an r.m.s. deviation of 0.587 and 0.403 Å, respectively, Fig. 8 ▸(*a*) and (*b*). The result clearly demonstrates the influence of solvent mol­ecule upon the mol­ecular packing in (I).

**Figure 9:**
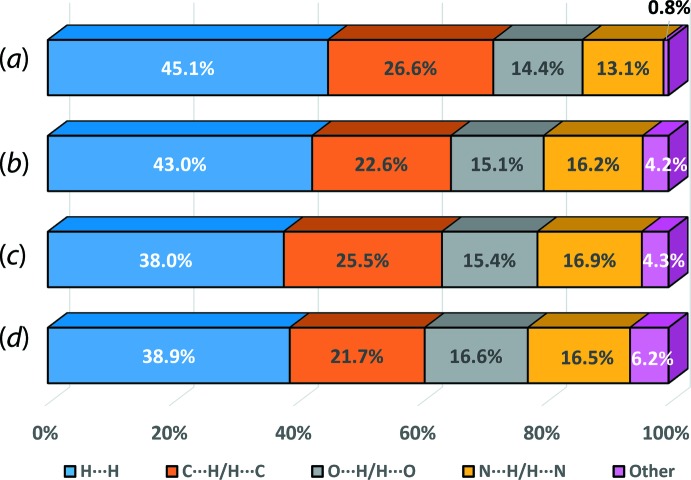
Percentage distribution of the corresponding close contacts on the Hirshfeld surfaces of ^4^ *L*H_2_ in (*a*) (I), (*b*) Form I – first independent mol­ecule, (*c*) Form I – second independent mol­ecule and (*d*) Form II.

Finally, and referring to Fig. 9 ▸, (I) and the two polymorphic forms of ^4^ *L*H_2_ exhibit a close similarity in the distribution of mol­ecular contacts as judged from the percentage contribution of the corresponding contacts on the Hirshfeld surface. The maximum variation in the distribution of H⋯H, H⋯C/C⋯H, H⋯O/O⋯H and H⋯N/N⋯H contacts ranged from 7.1, 4.9, 2.2 and 3.8%, respectively among the three crystals.

## Database survey   

**Figure 10:**
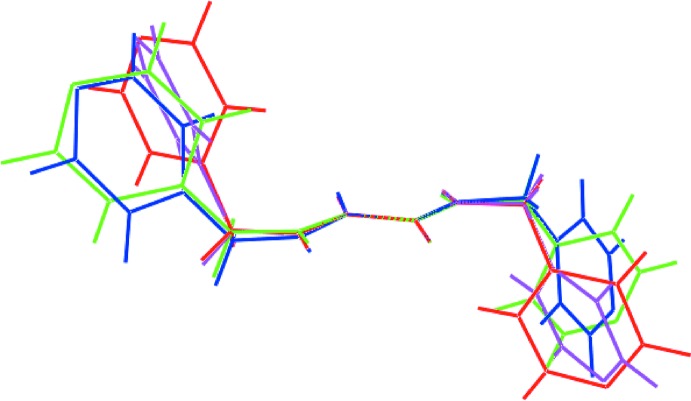
Overlay diagram for ^4^ *L*H_2_ mol­ecules in Form I – mol­ecule *a* (green image), Form I – mol­ecule *b* (blue), Form II (pink) and benzene solvate (red).

As mentioned in the *Chemical Context*, there are two polymorphs available for ^4^ *L*H_2_ (Lee & Wang, 2007 ▸; Lee, 2010 ▸). In Form I (Lee & Wang, 2007 ▸), two independent mol­ecules comprise the asymmetric unit whereas in Form II (Lee, 2010 ▸), half a centrosymmetric mol­ecule comprises the asymmetric unit. Selected geometric parameters for the polymorphs and (I) are given in Table 3 ▸. To a first approximation, the mol­ecular structures present the same geometric features, *i.e*. a planar central region and an anti­periplanar relationship between the pyridyl rings. It is noted that the central C—C bond is relatively long, a consistent observation traced to the influence of electronegative carbonyl-O and amide-N substituents and confirmed by DFT calculations in the case of polymorphic ^3^ *L*H_2_ (Jotani *et al.*, 2016 ▸) and in the sulfur analogues of ^3^ *L*H_2_, *i.e*. (*n*-C_5_H_4_N)CH_2_N(H)C(=S)C(=S)N(H)CH_2_(C_5_H_4_N-*n*), for *n* = 2, 3 and 4 (Zukerman-Schpector *et al.*, 2015 ▸). The similarity between the four mol­ecules of ^4^ *L*H_2_ in its polymorphs and benzene solvate are highlighted in Fig. 10 ▸.

## Synthesis and crystallization   

The precursor, *N*,*N*′-bis­(pyridin-4-ylmeth­yl)oxalamide, was prepared in accordance with the literature procedure (m.p. 486.3–487.6 K; lit. 486–487 K; Nguyen *et al.*, 1998 ▸): it (0.0015 g) was dissolved in DMF (0.5 ml) and then carefully layered in different experiments with 2 ml of benzene, *o*-xylene, *m*-xylene, *p*-xylene, toluene, pyridine and cyclo­hexane. Among these solvent systems, only the DMF–benzene mixture resulted in colourless crystals of the benzene solvate, (I); m.p. 411.4–413.7 K. IR (cm^−1^): 3322 ν(N—H), 3141–2804 ν(C—H), 1696–1661 ν(C=O), 1563–1515 ν(C=C), 1414 ν(C—N), 794 δ(C=C).

## Refinement   

Crystal data, data collection and structure refinement details are summarized in Table 4 ▸. The carbon-bound H atoms were placed in calculated positions (C—H = 0.95–0.99 Å) and were included in the refinement in the riding-model approximation, with *U*_iso_(H) set to 1.2*U*_eq_(C). The nitro­gen-bound H atom was located from difference-Fourier maps and refined with N—H = 0.88±0.01 Å, and with *U*_iso_(H) set to 1.2*U*_eq_(N).

## Supplementary Material

Crystal structure: contains datablock(s) I, global. DOI: 10.1107/S2056989019009551/hb7835sup1.cif

Structure factors: contains datablock(s) I. DOI: 10.1107/S2056989019009551/hb7835Isup2.hkl

Supporting information file. DOI: 10.1107/S2056989019009551/hb7835Isup3.cml

CCDC reference: 1938031

Additional supporting information:  crystallographic information; 3D view; checkCIF report

## Figures and Tables

**Table 1:** Hydrogen-bond geometry (Å, °) *Cg*1 is the centroid of the centrosymmetric (C11–C13,C11^i^–C13^i^) ring. *Cg*2 is the ring centroid of the (N1, C2–C5) ring.

*D*—H⋯*A*	*D*—H	H⋯*A*	*D*⋯*A*	*D*—H⋯*A*
N8—H8*N*⋯O10^i^	0.89 (1)	2.36 (1)	2.7129 (11)	104 (1)
N8—H8*N*⋯N1^ii^	0.89 (1)	2.03 (1)	2.8737 (12)	159 (1)
C7—H7*B*⋯*Cg*1	0.99	2.62	3.4037 (11)	136
C11—H11⋯*Cg*2^iii^	0.95	2.90	3.6361 (11)	136

Symmetry codes: (i) 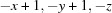; (ii) 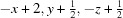; (iii) 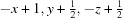.

**Table 2:** Inter­action energies (kJ mol^−1^) for selected close contacts

Close contact	*E*_electrostatic_	*E*_polarization_	*E*_dispersion_	*E*_exchange-repulsion_	*E*_total_	Symmetry operation
N8—H8⋯N1	−45.0	−12.2	−17.5	54.7	−38.1	−*x* + 2, *y* + 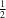 , −*z* + 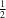
C7—H7*B*⋯*Cg*(benzene)	−10.1	−2.1	−23.7	22.6	−18.9	*x*, *y*, *z*
C11—H11⋯*Cg*(pyrid­yl)	−5.2	−1.1	−15.3	4.4	−16.9	−*x* + 1, *y* + 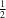 , −*z* + 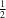

**Table 3:** Selected geometric data (Å, °) for mol­ecules of ^4^ *L*H_2_

Crystal	*Z*	central-C—C-central	C_2_N_2_O_2_/C_5_H_4_N	C_2_N_2_O_2_/C_5_H_4_N	Reference
Form I – mol­ecule *a*	2	1.541 (3)	84.59 (6) & 80.33 (4)	4.90 (6)	Lee & Wang (2007 ▸)
Form I – mol­ecule *b*		1.541 (3)	70.20 (5) & 68.01 (5)	6.68 (6)	
Form II	0.5	1.532 (2)	74.78 (4)	0	Lee (2010 ▸)
Benzene solvate (I)	0.5	1.5406 (18)	86.89 (3)	0	This work

**Table 4:** Experimental details

Crystal data	
Chemical formula	C_14_H_14_N_4_O_2_·C_6_H_6_
*M*_r_	348.40
Crystal system, space group	Monoclinic, *P*2_1_/*c*
Temperature (K)	100
*a*, *b*, *c* (Å)	5.80832 (8), 12.6437 (2), 12.1803 (2)
β (°)	99.942 (1)
*V* (Å^3^)	881.07 (2)
*Z*	2
Radiation type	Cu *K*α
μ (mm^−1^)	0.71
Crystal size (mm)	0.27 × 0.22 × 0.16

Data collection	
Diffractometer	Rigaku Oxford Diffraction SuperNova, Dual, Cu at zero, AtlasS2
Absorption correction	Multi-scan (*CrysAlis PRO*; Rigaku OD, 2015 ▸)
*T*_min_, *T*_max_	0.917, 1.000
No. of measured, independent and observed [*I* > 2σ(*I*)] reflections	7547, 1838, 1741
*R*_int_	0.018
(sin θ/λ)_max_ (Å^−1^)	0.630

Refinement	
*R*[*F* ^2^ > 2σ(*F* ^2^)], *wR*(*F* ^2^), *S*	0.034, 0.092, 1.03
No. of reflections	1838
No. of parameters	121
No. of restraints	1
H-atom treatment	H atoms treated by a mixture of independent and constrained refinement
Δρ_max_, Δρ_min_ (e Å^−3^)	0.26, −0.22

Computer programs: *CrysAlis PRO* (Rigaku OD, 2015 ▸), *SHELXT* (Sheldrick, 2015*a* ▸), *SHELXL2018* (Sheldrick, 2015*b* ▸), *ORTEP-3 for Windows* (Farrugia, 2012 ▸), *OLEX2* (Dolomanov *et al.*, 2009 ▸), *Mercury* (Macrae *et al.*, 2006 ▸), *DIAMOND* (Brandenburg, 2006 ▸) and *QMol* (Gans & Shalloway, 2001 ▸) and *publCIF* (Westrip, 2010 ▸).

## supplementary crystallographic information

### Crystal data

**Table:**

C_14_H_14_N_4_O_2_·C_6_H_6_	*F*(000) = 368
*M**_r_* = 348.40	*D*_x_ = 1.313 Mg m^−^^3^
Monoclinic, *P*2_1_/*c*	Cu *K*α radiation, λ = 1.54184 Å
*a* = 5.80832 (8) Å	Cell parameters from 5470 reflections
*b* = 12.6437 (2) Å	θ = 3.7–76.1°
*c* = 12.1803 (2) Å	µ = 0.71 mm^−^^1^
β = 99.942 (1)°	*T* = 100 K
*V* = 881.07 (2) Å^3^	Block, colourless
*Z* = 2	0.27 × 0.22 × 0.16 mm

### Data collection

**Table:**

Rigaku Oxford Diffraction SuperNova, Dual, Cu at zero, AtlasS2 diffractometer	1838 independent reflections
Radiation source: micro-focus sealed X-ray tube, SuperNova (Cu) X-ray Source	1741 reflections with *I* > 2σ(*I*)
Mirror monochromator	*R*_int_ = 0.018
Detector resolution: 5.2303 pixels mm^-1^	θ_max_ = 76.3°, θ_min_ = 5.1°
ω scans	*h* = −7→6
Absorption correction: multi-scan (CrysAlis PRO; Rigaku OD, 2015)	*k* = −15→15
*T*_min_ = 0.917, *T*_max_ = 1.000	*l* = −14→15
7547 measured reflections	

### Refinement

**Table:**

Refinement on *F*^2^	Primary atom site location: structure-invariant direct methods
Least-squares matrix: full	Secondary atom site location: difference Fourier map
*R*[*F*^2^ > 2σ(*F*^2^)] = 0.034	Hydrogen site location: mixed
*wR*(*F*^2^) = 0.092	H atoms treated by a mixture of independent and constrained refinement
*S* = 1.03	*w* = 1/[σ^2^(*F*_o_^2^) + (0.052*P*)^2^ + 0.2834*P*] where *P* = (*F*_o_^2^ + 2*F*_c_^2^)/3
1838 reflections	(Δ/σ)_max_ < 0.001
121 parameters	Δρ_max_ = 0.26 e Å^−^^3^
1 restraint	Δρ_min_ = −0.22 e Å^−^^3^

### Special details

**Table:**

Geometry. All esds (except the esd in the dihedral angle between two l.s. planes) are estimated using the full covariance matrix. The cell esds are taken into account individually in the estimation of esds in distances, angles and torsion angles; correlations between esds in cell parameters are only used when they are defined by crystal symmetry. An approximate (isotropic) treatment of cell esds is used for estimating esds involving l.s. planes.

### Fractional atomic coordinates and isotropic or equivalent isotropic displacement parameters (Å^2^)

**Table:**

	*x*	*y*	*z*	*U*_iso_*/*U*_eq_	
O10	0.38046 (13)	0.37705 (6)	0.02822 (6)	0.02248 (19)	
N1	1.08230 (15)	0.16191 (7)	0.27845 (7)	0.0201 (2)	
N8	0.60308 (14)	0.49128 (6)	0.14611 (7)	0.01478 (19)	
H8N	0.681 (2)	0.5518 (8)	0.1539 (10)	0.018*	
C2	0.90416 (19)	0.16751 (8)	0.33490 (9)	0.0212 (2)	
H2	0.886428	0.111791	0.385095	0.025*	
C3	0.74433 (18)	0.24969 (8)	0.32443 (8)	0.0182 (2)	
H3	0.619613	0.249378	0.365660	0.022*	
C4	0.76959 (16)	0.33301 (7)	0.25225 (8)	0.0146 (2)	
C5	0.95261 (17)	0.32732 (8)	0.19256 (8)	0.0166 (2)	
H5	0.974925	0.381952	0.141884	0.020*	
C6	1.10251 (17)	0.24105 (8)	0.20768 (8)	0.0179 (2)	
H6	1.225774	0.237994	0.165662	0.022*	
C7	0.60511 (16)	0.42643 (8)	0.24442 (8)	0.0154 (2)	
H7A	0.444777	0.400031	0.244779	0.018*	
H7B	0.649954	0.471177	0.311395	0.018*	
C9	0.49063 (16)	0.46013 (7)	0.04685 (8)	0.0152 (2)	
C11	0.4304 (2)	0.59937 (9)	0.45845 (8)	0.0238 (2)	
H11	0.382675	0.667329	0.429930	0.029*	
C12	0.27389 (19)	0.51554 (9)	0.44356 (9)	0.0244 (2)	
H12	0.119021	0.526258	0.404969	0.029*	
C13	0.3429 (2)	0.41613 (9)	0.48487 (9)	0.0240 (2)	
H13	0.235695	0.358784	0.474473	0.029*	

### Atomic displacement parameters (Å^2^)

**Table:**

	*U*^11^	*U*^22^	*U*^33^	*U*^12^	*U*^13^	*U*^23^
O10	0.0285 (4)	0.0190 (4)	0.0182 (4)	−0.0090 (3)	−0.0007 (3)	0.0018 (3)
N1	0.0205 (4)	0.0181 (4)	0.0208 (4)	0.0028 (3)	0.0012 (3)	0.0003 (3)
N8	0.0163 (4)	0.0123 (4)	0.0153 (4)	−0.0007 (3)	0.0016 (3)	0.0012 (3)
C2	0.0249 (5)	0.0181 (5)	0.0202 (5)	0.0010 (4)	0.0027 (4)	0.0047 (4)
C3	0.0195 (5)	0.0190 (5)	0.0166 (5)	−0.0002 (4)	0.0041 (4)	0.0013 (4)
C4	0.0152 (4)	0.0151 (5)	0.0126 (4)	−0.0010 (3)	−0.0007 (3)	−0.0016 (3)
C5	0.0174 (5)	0.0164 (5)	0.0156 (5)	−0.0016 (4)	0.0019 (4)	0.0005 (3)
C6	0.0168 (5)	0.0191 (5)	0.0177 (5)	−0.0001 (4)	0.0024 (4)	−0.0019 (4)
C7	0.0166 (4)	0.0161 (5)	0.0136 (4)	0.0008 (3)	0.0031 (3)	0.0003 (3)
C9	0.0146 (4)	0.0148 (5)	0.0161 (5)	0.0011 (3)	0.0025 (4)	0.0012 (4)
C11	0.0337 (6)	0.0228 (5)	0.0169 (5)	0.0095 (4)	0.0098 (4)	0.0043 (4)
C12	0.0201 (5)	0.0372 (6)	0.0164 (5)	0.0070 (4)	0.0050 (4)	0.0025 (4)
C13	0.0289 (6)	0.0275 (6)	0.0175 (5)	−0.0041 (4)	0.0091 (4)	−0.0014 (4)

### Geometric parameters (Å, º)

**Table:**

O10—C9	1.2305 (12)	C5—C6	1.3879 (14)
N1—C2	1.3394 (14)	C5—H5	0.9500
N1—C6	1.3391 (13)	C6—H6	0.9500
N8—C9	1.3307 (13)	C7—H7A	0.9900
N8—C7	1.4496 (12)	C7—H7B	0.9900
N8—H8N	0.886 (8)	C9—C9^i^	1.5406 (18)
C2—C3	1.3845 (14)	C11—C12	1.3876 (17)
C2—H2	0.9500	C11—C13^ii^	1.3911 (16)
C3—C4	1.3961 (14)	C11—H11	0.9500
C3—H3	0.9500	C12—C13	1.3871 (16)
C4—C5	1.3895 (14)	C12—H12	0.9500
C4—C7	1.5118 (13)	C13—H13	0.9500
			
C2—N1—C6	116.89 (9)	N8—C7—C4	114.14 (8)
C9—N8—C7	121.05 (8)	N8—C7—H7A	108.7
C9—N8—H8N	121.0 (8)	C4—C7—H7A	108.7
C7—N8—H8N	118.0 (8)	N8—C7—H7B	108.7
N1—C2—C3	123.89 (9)	C4—C7—H7B	108.7
N1—C2—H2	118.1	H7A—C7—H7B	107.6
C3—C2—H2	118.1	O10—C9—N8	125.33 (9)
C2—C3—C4	118.86 (9)	O10—C9—C9^i^	121.53 (11)
C2—C3—H3	120.6	N8—C9—C9^i^	113.14 (10)
C4—C3—H3	120.6	C12—C11—C13^ii^	119.98 (10)
C5—C4—C3	117.62 (9)	C12—C11—H11	120.0
C5—C4—C7	122.66 (9)	C13^ii^—C11—H11	120.0
C3—C4—C7	119.70 (9)	C13—C12—C11	120.20 (10)
C4—C5—C6	119.36 (9)	C13—C12—H12	119.9
C4—C5—H5	120.3	C11—C12—H12	119.9
C6—C5—H5	120.3	C12—C13—C11^ii^	119.82 (11)
N1—C6—C5	123.37 (9)	C12—C13—H13	120.1
N1—C6—H6	118.3	C11^ii^—C13—H13	120.1
C5—C6—H6	118.3		
			
C6—N1—C2—C3	−0.44 (15)	C9—N8—C7—C4	76.76 (11)
N1—C2—C3—C4	−0.91 (16)	C5—C4—C7—N8	19.14 (13)
C2—C3—C4—C5	1.46 (14)	C3—C4—C7—N8	−162.91 (8)
C2—C3—C4—C7	−176.60 (9)	C7—N8—C9—O10	0.23 (15)
C3—C4—C5—C6	−0.74 (14)	C7—N8—C9—C9^i^	−179.96 (9)
C7—C4—C5—C6	177.26 (8)	C13^ii^—C11—C12—C13	−0.13 (17)
C2—N1—C6—C5	1.23 (15)	C11—C12—C13—C11^ii^	0.13 (17)
C4—C5—C6—N1	−0.64 (15)		

Symmetry codes: (i) −*x*+1, −*y*+1, −*z*; (ii) −*x*+1, −*y*+1, −*z*+1.

### Hydrogen-bond geometry (Å, º)

*Cg*1 is the centroid of the centrosymmetric (C11–C13,C11^i^–C13^i^) ring. *Cg*2 is the ring centroid of the (N1, C2–C5) ring.

**Table:**

*D*—H···*A*	*D*—H	H···*A*	*D*···*A*	*D*—H···*A*
N8—H8*N*···O10^i^	0.89 (1)	2.36 (1)	2.7129 (11)	104 (1)
N8—H8*N*···N1^iii^	0.89 (1)	2.03 (1)	2.8737 (12)	159 (1)
C7—H7*B*···*Cg*1	0.99	2.62	3.4037 (11)	136
C11—H11···*Cg*2^iv^	0.95	2.90	3.6361 (11)	136

Symmetry codes: (i) −*x*+1, −*y*+1, −*z*; (iii) −*x*+2, *y*+1/2, −*z*+1/2; (iv) −*x*+1, *y*+1/2, −*z*+1/2.
